# Comparative analysis of percutaneous aspiration and percutaneous catheter drainage for the treatment of liver abscess: A systematic review and meta-analysis

**DOI:** 10.1097/MD.0000000000044465

**Published:** 2025-09-12

**Authors:** Xu-Feng Li, Dan-Mei Pan

**Affiliations:** a Department of Infectious Disease, Haiyan People’s Hospital, Haiyan County, Jiaxing City, Zhejiang Province, China; b Department of Infectious Disease, Ningbo No. 2 Hospital, Haishu District, Ningbo City, Zhejiang Province, China.

**Keywords:** liver abscess, meta-analysis, percutaneous aspiration, percutaneous catheter drainage, treatment outcomes

## Abstract

**Background:**

Liver abscesses are a serious clinical condition requiring effective treatment to minimize complications and improve outcomes. Percutaneous aspiration (PA) and percutaneous catheter drainage (PCD) are the most common minimally invasive approaches for managing liver abscesses, yet their comparative efficacy and safety remain debated. This systematic review and meta-analysis aim to evaluate the effectiveness, safety, and recurrence outcomes of PA versus PCD.

**Methods:**

A comprehensive search was conducted in PubMed, Embase, Web of Science, and Cochrane Library, following Preferred Reporting Items for Systematic Reviews and Meta-Analyses guidelines. Studies were included if they compared PA and PCD in adult patients with liver abscesses, reported quantitative clinical outcomes, and met predefined inclusion criteria. Data extraction and quality assessment were performed independently by 2 reviewers. Statistical analyses were conducted using fixed-effects or random-effects models based on heterogeneity. Sensitivity analyses and funnel plots were employed to assess the robustness and publication bias of the findings.

**Results:**

A total of 12 studies with 1290 participants were included. PCD demonstrated significantly higher treatment success rates than PA (RR = 1.19, 95% CI [1.10, 1.29], *P* < .001) and shorter time to clinical improvement (weighted mean difference = −1.92 days, 95% CI [−2.71, −1.13], *P* < .001). PCD also showed a lower 6-month recurrence rate (RR = 0.44, 95% CI [0.26, 0.75], *P* < .001). No significant differences were observed in complication rates between PA and PCD (RR = 1.13, 95% CI [0.45, 2.85], *P* > .05). Funnel plots revealed no significant publication bias.

**Conclusions:**

This meta-analysis demonstrates that PCD offers superior outcomes over PA for treating liver abscesses, including higher success rates, faster recovery, and lower recurrence, with comparable safety profiles. Further high-quality randomized controlled trials are needed to validate these findings and refine clinical guidelines.

## 1. Introduction

Liver abscess, a potentially life-threatening condition, is defined as a localized collection of pus within the liver parenchyma, most commonly caused by bacterial (especially *Klebsiella pneumoniae* or *Escherichia coli*), fungal, or parasitic infections such as *Entamoeba histolytica*.^[1,2]^ It typically presents with nonspecific clinical symptoms, including fever, right upper quadrant abdominal pain, and malaise, which may contribute to delayed diagnosis and treatment.^[3]^ Despite advancements in diagnostic imaging (e.g., ultrasonography, computed tomography) and microbiological techniques (e.g., culture, polymerase chain reaction), the clinical management of liver abscess remains challenging due to the variability in etiology, abscess characteristics, and host response.^[3,4]^ Epidemiologically, the incidence of liver abscess varies geographically. In developing regions, amebic liver abscess is more prevalent due to endemic parasitic infections, whereas pyogenic liver abscess is more common in developed countries and frequently associated with comorbidities such as diabetes mellitus, malignancy, or immunosuppression. Historically, open surgical drainage was the standard of care. However, with the advent of minimally invasive techniques, percutaneous drainage methods have become the cornerstone of treatment.^[5–7]^

Among these, percutaneous aspiration (PA) and percutaneous catheter drainage (PCD) are the 2 primary image-guided interventions. PA involves inserting a fine needle into the abscess cavity under imaging guidance to aspirate pus, typically in a single session.^[5,8]^ It is considered less invasive, cost-effective, and is often suitable for small, unilocular abscesses with low-viscosity contents. However, PA may be inadequate for larger or multiloculated abscesses and frequently requires multiple procedures for complete resolution.^[9,10]^ In contrast, PCD entails the placement of a catheter into the abscess cavity, enabling continuous drainage over several days. This approach is generally preferred for larger abscesses (>5 cm), multiloculated lesions, or those at risk of rupture. While PCD is technically more complex and may be associated with catheter-related complications, it has been linked to higher success rates, shorter hospitalization, and lower recurrence in many studies.^[11,12]^ Despite widespread clinical use, the comparative efficacy and safety of PA versus PCD remain subjects of ongoing debate, and existing studies have yielded inconsistent findings due to small sample sizes and heterogeneity in study design and patient characteristics.^[13,14]^

Therefore, a comprehensive synthesis of the current literature is warranted. This systematic review and meta-analysis aim to compare the clinical efficacy, safety, and recurrence outcomes of PA and PCD in the management of liver abscess. The findings are intended to inform clinical decision-making and contribute to the development of standardized treatment protocols for this condition.

## 2. Methods

### 2.1. Search strategy

The search strategy for this meta-analysis adhered to the Preferred Reporting Items for Systematic Reviews and Meta-Analyses guidelines.^[15]^ We systematically searched 4 electronic databases (PubMed, Embase, Web of Science, and Cochrane Library) on September 26, 2024, without applying any time restrictions. The search terms included a combination of keywords and Boolean operators: (needles OR catheterization OR drainage OR catheter) AND (“liver abscess*” OR “hepatic abscess”). These terms were carefully selected to align with the PICO framework (Patient, Intervention, Comparison, Outcome), ensuring a thorough and comprehensive retrieval of relevant studies. No language restrictions were applied to the search. Additionally, reference lists of relevant articles were manually screened to identify any additional records that may not have been captured through the database searches.

### 2.2. Inclusion criteria and exclusion criteria

The inclusion criteria for this meta-analysis were as follows: studies directly comparing PA and PCD for the treatment of liver abscess; Only randomized controlled trials (RCTs) that reported quantitative data on clinical outcomes, including treatment success rates, complication rates, recurrence rates, or hospital stay durations, were included; studies conducted on adult patients (≥18 years old) with a confirmed diagnosis of liver abscess, irrespective of etiology; studies published in peer-reviewed journals and accessible in full-text format; and studies available in any language. The exclusion criteria included studies that did not include a direct comparison between PA and PCD, case reports, review articles, conference abstracts, or studies with insufficient data for extraction. Additionally, studies focusing on pediatric populations or animals, duplicate publications, or studies with overlapping data sets were excluded.

### 2.3. Literature screening and data extraction

Data extraction was conducted independently by 2 reviewers, with all results cross-checked for consistency. Any discrepancies identified during the process were resolved through discussion between the reviewers, with consultation from a third-party reviewer if necessary. The extracted data included the following variables: country of study, total number of participants, type of abscess (pyogenic, amebic, or both), abscess size, antibiotics used, maximum number of needle aspirations, needle caliber and type, catheter caliber and type, follow-up duration, and the definition of success rate.

### 2.4. Quality assessment

The methodological quality of the included studies was evaluated using the Cochrane Collaboration’s risk of bias tool.^[16]^ Two reviewers independently assessed several key domains, including the methods for random sequence generation, allocation concealment, blinding of participants and personnel, handling of incomplete outcome data, selective reporting, and any additional sources of potential bias. Each domain was classified as having a low, high, or unclear risk of bias. Any discrepancies between the reviewers were addressed through discussion, with the involvement of a third reviewer when consensus could not be reached.

### 2.5. Statistical analyses

The heterogeneity among the included studies was assessed using Chi-square statistics and quantified by the *I*^2^ value. If the *I*^2^ value was <50% and the corresponding *P*-value was greater than or equal to .10, it indicated no significant heterogeneity, and a fixed-effect model was used to calculate the pooled effect size. Conversely, when the *I*^2^ value was equal to or >50% or the corresponding *P*-value was <.10, significant heterogeneity was assumed, and a random-effects model was applied to estimate the combined effect size. Sensitivity analyses were conducted to assess the robustness of the results and identify the potential influence of individual studies on the pooled effect size. This was performed by sequentially excluding each study from the analysis and recalculating the overall effect size. Additionally, potential sources of heterogeneity were explored to ensure reliability. Publication bias was assessed using both visual inspection of funnel plot symmetry and Egger regression test. A symmetrical funnel plot and a nonsignificant result from Egger test (*P* > .05) were interpreted as indicating no substantial publication bias. All statistical tests were 2-sided, with a *P*-value of <.05 considered statistically significant. Data analysis was performed using Stata version 17 (StataCorp, College Station).

## 3. Results

### 3.1. Search results and study selection

**Figure 1.**
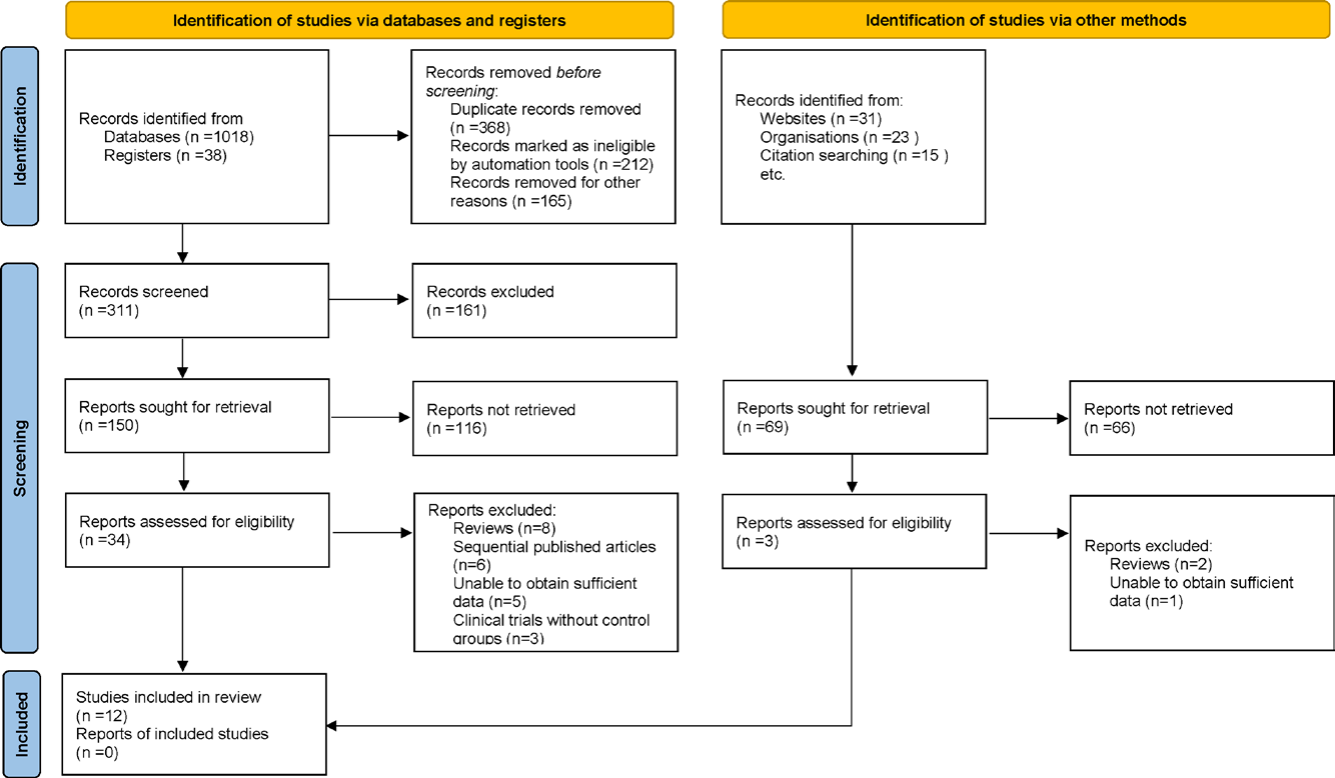
Flowchart of study selection process.

At the outset of this systematic review and meta-analysis, a comprehensive search across multiple electronic databases initially yielded 1125 potentially relevant articles. Duplicates were removed to ensure each study was represented only once. Titles and abstracts were then reviewed according to predefined inclusion and exclusion criteria, which considered study methodology, population demographics, measured clinical outcomes, and research quality. After this initial screening, 37 articles were selected for full-text review. Following a detailed evaluation by multiple independent investigators, 25 articles were excluded for the following reasons: review articles (n = 10), sequential publications (n = 6), insufficient data (n = 6), and clinical trials without control groups (n = 3). Ultimately, 12 studies met all inclusion criteria and were included in the final meta-analysis^[11,17–27]^ (Fig. 1).

### 3.2. Study characteristics

The studies included in this meta-analysis were primarily conducted in India, with a few studies originating from Egypt and other countries. A total of 1425 participants were involved across all studies. The studies predominantly focused on patients with abscesses larger than 5 cm, with a mix of both pyogenic and amebic abscesses, although a few studies specifically focused on pyogenic abscesses. The antibiotic regimens used in the studies varied, but commonly included ceftriaxone, metronidazole, and other antibiotics such as cefazolin and augmentin. Needle aspiration was a common intervention across the studies, with needle sizes typically ranging from 16 to 18 gauge, although some studies used different needle types, such as 23 gauge or 18 gauge trocar needles. The catheter types were mostly 12 to 14 French pigtail catheters, and in some cases, plastic-based or multi-sidehole pigtail catheters were used. The follow-up durations across studies ranged from 4 months to 2 years, with most studies following up for 6 months. Success rates were generally defined by clinical and sonographic resolution of the abscess or sufficient drainage leading to infection resolution and discharge from the hospital. However, a few studies did not provide a clear definition of success or used alternative success criteria (Table 1).

**Table 1:** Characteristics of studies included in the meta-analysis.

Study author	Year	Country	Total participants	Size of abscess (cm)	Needle caliber and type	Type of abscess	Used antibiotics	Catheter caliber and Type	Follow-up duration	Success rate definition
Darshan et al^[19]^	2022	India	50	>5	16–18 gauge long needle	Amoebic and pyogenic	Ceftriaxone 1 g 12 hourly, metronidazole 500 mg 6 hourly	14 French pigtail catheter with sharp trocar	6 mo	Not applicable (N/A)
Ahmed et al^[18]^	2021	India	543	>5	16–18 gauge long needle	Amoebic and pyogenic	Ceftriaxone 1 g 12 hourly, metronidazole 500 mg 6 hourly	14 French pigtail catheter	6 mo	Not applicable (N/A)
Surya et al^[26]^	2020	India	100	>5	16 gauge long needle	Amoebic and pyogenic	Ceftriaxone 1 g IV every 12 h and metronidazole 500 mg IV every 6 h	14 French pigtail catheter	6 mo	Clinical and sonographic resolution
Kulhari and Mandia^[21]^	2019	India	190	>5	16–18 gauge long needle	Amoebic and pyogenic	N/A	12 French pigtail catheter	6 mo	Clinical and sonographic resolution
Singh et al^[24]^	2019	India	66	>3	18 gauge disposable trocar needle	Amoebic and pyogenic	N/A	12 French pigtail catheter	6 mo	Sufficient drainage without surgical drainage leading to infection resolution and discharge from hospital
Abusedera and El-Badry^[17]^	2014	Egypt	88	>2	18 gauge trocar needle	Pyogenic	Cefazoline 1 g/12 h, augmentin 1.2 g/8 h IV, metronidazole 500 mg IV or orally (3 times a day)	Plastic-based catheter	6 months	Clinical and sonographic resolution
Singh et al^[25]^	2013	India	60	>5	23 gauge needle	Amoebic and pyogenic	Metronidazole 750 mg IV every t.i.d., cefazolin 1 g IV b.i.d., gentamicin 80 mg IV b.i.d., chloroquine 600 mg for 2 d (split doses), followed by 300 mg for 19 days (split doses)	12 French pigtail catheter	6 mo	Clinical and sonographic resolution
Gupta et al^[20]^	2011	India	82	>10	16 gauge disposable trocar needle	Amoebic	Intravenous metronidazole for 10 d or until fever subsided, followed by oral metronidazole 40 mg/kg/d in divided doses for 3 wk	14 French multi-sidehole pigtail catheter	2 yr	Clinical and sonographic resolution
Singh et al^[23]^	2009	India	72	>10	16 gauge disposable trocar needle	Amoebic and pyogenic	Ceftriaxone 1 g, gentamicin 1 mg/kg, metronidazole 7.5 mg/kg, each administered 3 times a day	14 French multi-sidehole pigtail catheter	4 mo	Clinical and sonographic resolution
Zerem and Hadzic^[11]^	2007	Bosnia and Herzegovina	60	>3	18 gauge disposable trocar needle	Pyogenic	IV cefazolin 1 g 3 times a day, gentamicin 1 mg/kg 3 times a day for 10 days	8 French multiple sidehole pigtail catheter	6 mo	Clinical and sonographic resolution
Yu et al^[27]^	2004	China	64	>3	18 gauge disposable trocar needle	Pyogenic	Ampicillin 500 mg 6 hourly, cefuroxime 750 mg 8 hourly, metronidazole 500 mg 8 hourly	8 French multi-sidehole pigtail catheter	Biweekly until completion of oral antibiotics without recurrent infection	Sufficient drainage without surgical drainage leading to infection resolution and discharge from hospital
Rajak et al^[22]^	1998	India	50	N/A	18 Gauge needle	Amoebic and pyogenic	Broad spectrum antibiotics, including cloxacillin (150 mg/kg per day IV), gentamicin (4.5 mg/kg per day IV), metronidazole (500 mg IV or 800 mg orally 3 times a day), and chloroquine	8–12 French pigtail or malecot drainage catheter	8–37 weeks (mean, 20 weeks)	Clinical and sonographic resolution

NA = not available.

### 3.3. Results of quality assessment

**Figure 2.**
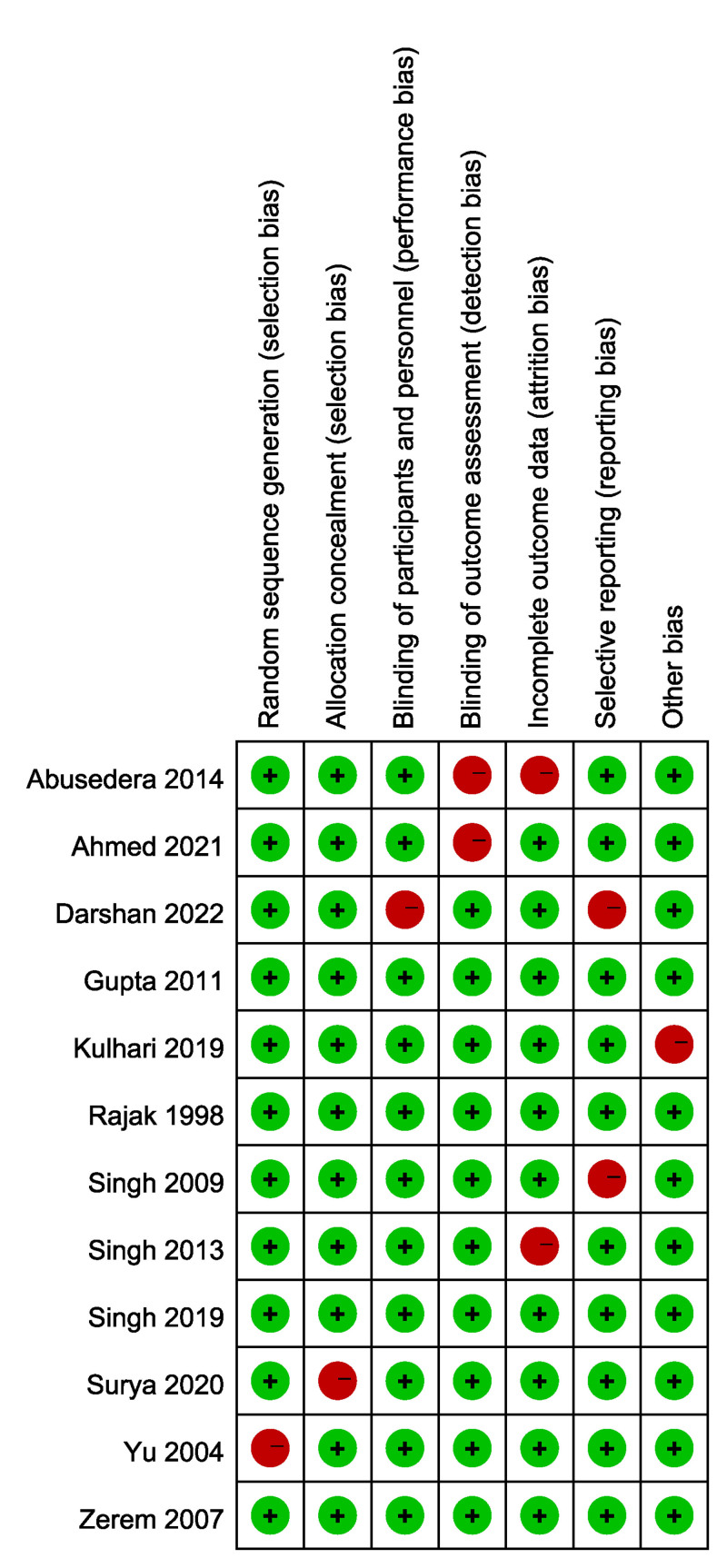
Risk of bias assessment of included studies based on Cochrane Collaboration’s criteria. Red represents high risk, and green indicates low risk.

The quality evaluation of the studies included in this meta-analysis, based on the Cochrane Collaboration’s risk of bias tool, reveals a generally high level of methodological rigor. A majority of the studies demonstrated a low risk of bias in areas such as random sequence generation and outcome assessment blinding, suggesting robust internal validity. However, some issues were identified in allocation concealment and selective reporting, with several studies showing high risks of bias. These concerns may stem from challenges in adhering to double-blind protocols in real-world clinical settings. Nevertheless, outcome assessment blinding was consistently implemented across the studies, contributing to the reliability of the findings (Fig. 2).

### 3.4. Success rates of percutaneous aspiration and percutaneous catheter drainage

**Figure 3.**
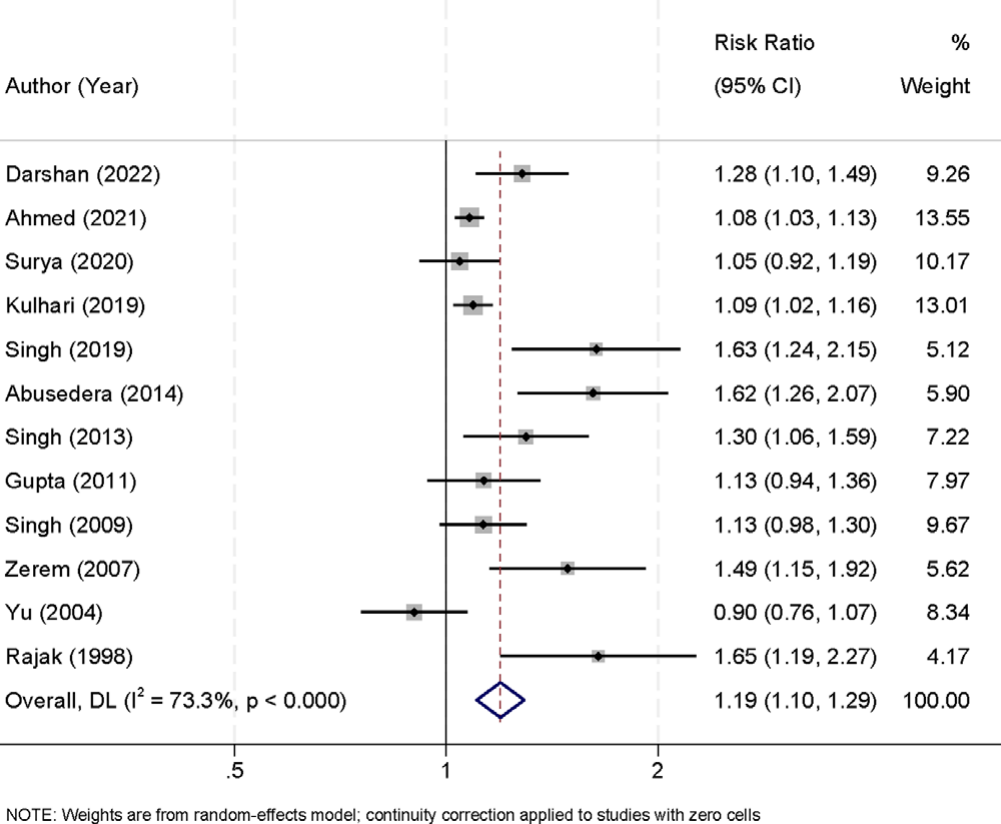
Forest plot of success rates comparing percutaneous aspiration and percutaneous catheter drainage.

This meta-analysis included 12 studies to compare the treatment success rates of PA and PCD for liver abscess management. A significant degree of heterogeneity was detected among the included studies (*I*^2^ = 73.3%, *P* < .001), prompting the application of a random-effects model to synthesize the data and account for variations between studies. The pooled results demonstrated that PCD had a markedly higher treatment success rate compared to PA. The calculated relative risk (RR) for successful treatment with PCD was 1.19 (95% CI [1.10, 1.29], *P* < .001), reflecting a 19% greater probability of achieving abscess resolution with PCD (Fig. 3). This result highlights the clinical superiority of PCD, particularly in addressing cases involving larger abscesses, multiloculated structures, or recurrent infections, where PA may be less effective.

**Figure 4.**
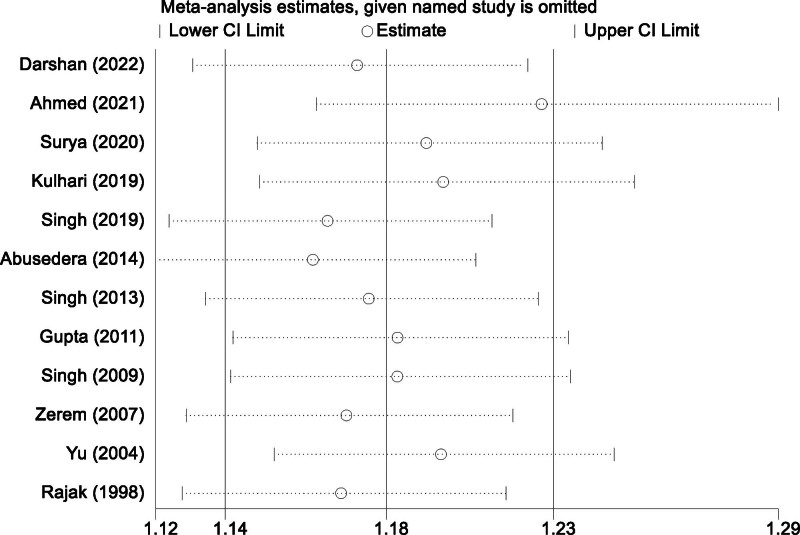
Sensitivity analysis for success rates of percutaneous aspiration versus percutaneous catheter drainage.

The substantial heterogeneity observed among the studies can be attributed to differences in several factors, including study design, patient demographics, abscess characteristics, and procedural protocols. Variations in abscess size, unilocular versus multiloculated configurations, and operator skill levels may have contributed to the observed discrepancies in treatment outcomes across the studies. To address these concerns, a sensitivity analysis was conducted by systematically excluding individual studies. The results of this analysis confirmed that the pooled effect remained consistent and robust regardless of the exclusion of any single study (Fig. 4). This stability indicates that no individual study disproportionately influenced the overall findings, enhancing the reliability and validity of the meta-analysis results.

### 3.5. Time to clinical improvement with percutaneous aspiration and percutaneous catheter drainage

**Figure 5.**
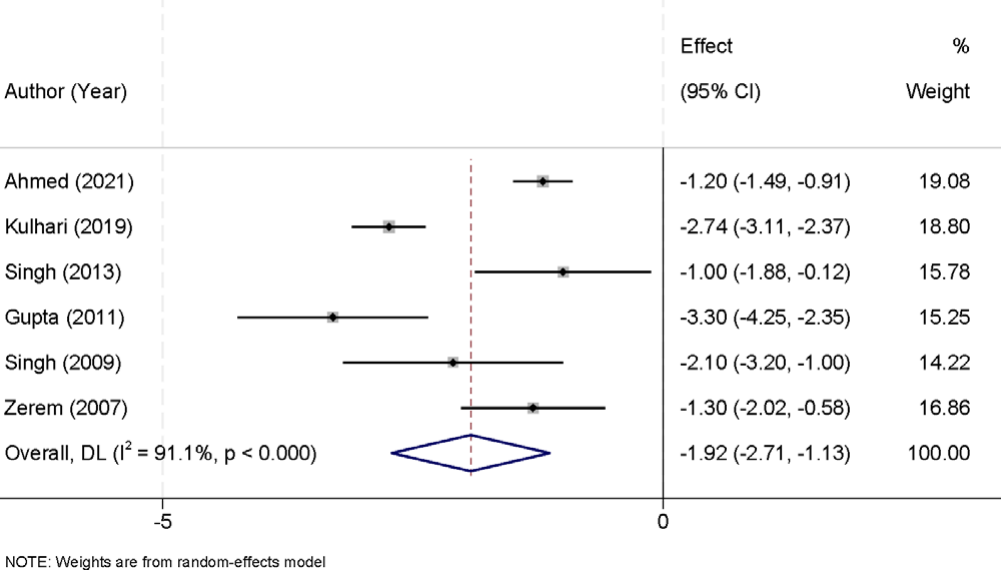
Forest plot of time to clinical improvement comparing percutaneous aspiration and percutaneous catheter drainage.

**Figure 6.**
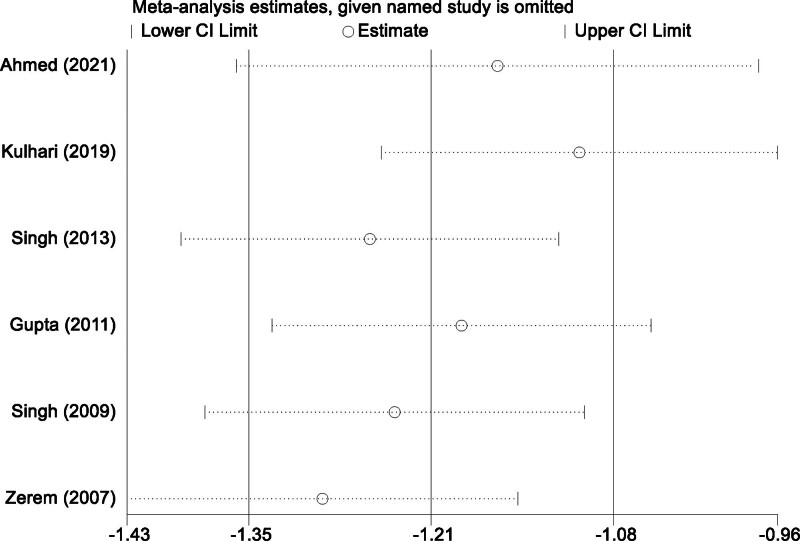
Sensitivity analysis for time to clinical improvement with percutaneous aspiration and percutaneous catheter drainage.

A total of 6 studies were included in this meta-analysis to compare the time to clinical improvement between PA and PCD in the management of liver abscesses. Significant heterogeneity was observed among the studies (*I*^2^ = 91.1%, *P* < .001), and a random-effects model was applied to synthesize the data, accounting for the variability across studies. The pooled analysis indicated that PCD was associated with a significantly shorter time to clinical improvement compared to PA. The weighted mean difference (MD) was −1.92 days (95% CI [−2.71, −1.13], *P* < .001), demonstrating that PCD expedited symptom relief by nearly 2 days compared to PA (Fig. 5). Sensitivity analysis confirmed the robustness of these findings, as the pooled effect remained consistent regardless of the exclusion of any individual study (Fig. 6). These results underscore the clinical advantage of PCD in facilitating faster recovery, highlighting its potential to reduce hospital stays and improve patient outcomes.

### 3.6. Recurrence after 6 months with percutaneous aspiration and percutaneous catheter drainage

**Figure 7.**
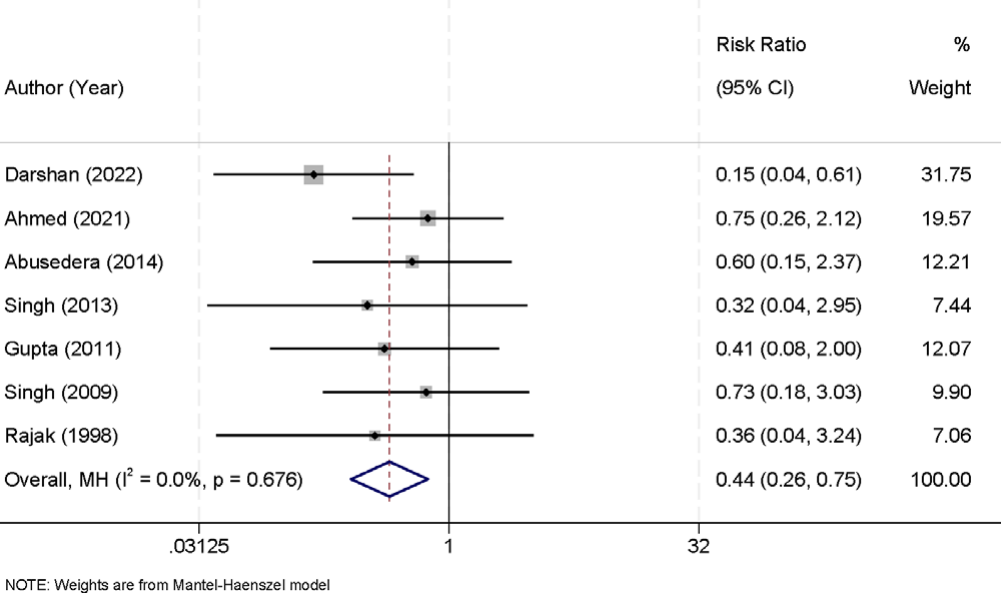
Forest plot of 6-month recurrence rates comparing percutaneous aspiration and percutaneous catheter drainage.

Seven studies were included in this meta-analysis to evaluate the recurrence rates of liver abscesses within 6 months following treatment with PA or PCD. No significant heterogeneity was observed among the studies (*I*^2^ = 0.0%, *P* = .676), allowing the use of a fixed-effects model to combine the results. The pooled analysis demonstrated that PCD was associated with a significantly lower recurrence rate compared to PA. The RR for recurrence with PCD was 0.44 (95% CI [0.26, 0.75], *P* < .001), indicating a 56% reduction in recurrence risk (Fig. 7). These findings highlight the long-term efficacy of PCD in reducing abscess recurrence, reinforcing its clinical utility as a preferred treatment strategy.

### 3.7. Complication rates of percutaneous aspiration versus percutaneous catheter drainage

**Figure 8.**
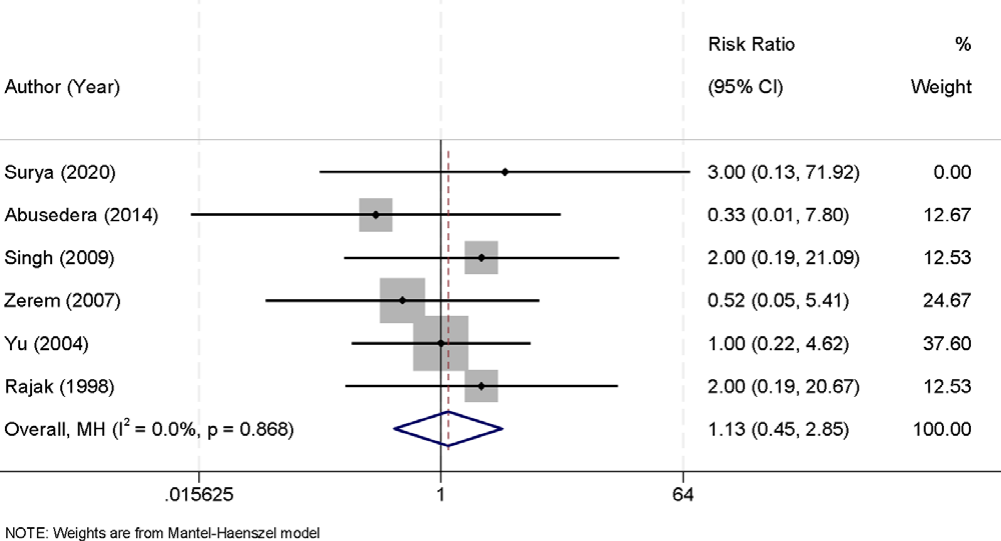
Forest plot of complication rates for percutaneous aspiration versus percutaneous catheter drainage.

Six studies were included in this meta-analysis to compare the complication rates associated with PA and PCD in the treatment of liver abscesses. The analysis revealed no significant heterogeneity among the included studies (*I*^2^ = 0.0%, *P* = .868), enabling the use of a fixed-effects model to pool the results. The findings indicated no significant difference in the overall complication rates between the 2 treatment methods. The RR was 1.13 (95% CI [0.45, 2.85], *P* > .05), suggesting comparable safety profiles for both PA and PCD (Fig. 8). These results provide evidence that both approaches are similarly safe and well-tolerated, allowing clinicians to prioritize other factors, such as efficacy and patient-specific considerations, when selecting a treatment strategy.

### 3.8. Assessment of publication bias

**Figure 9.**
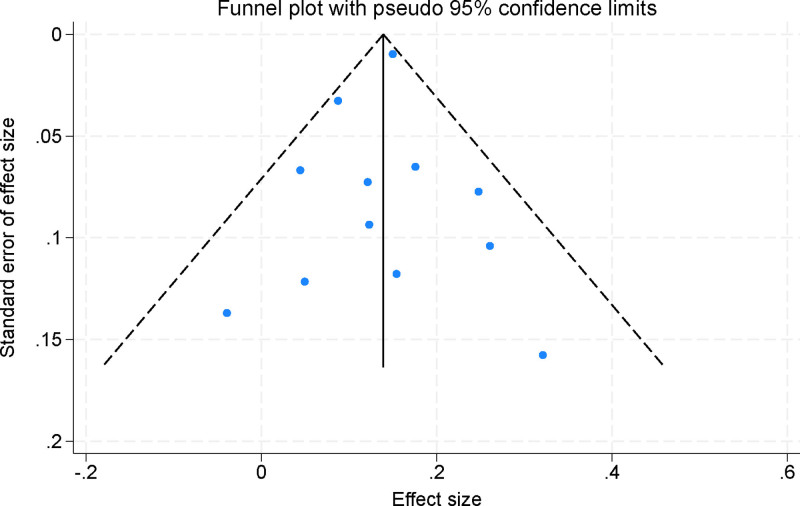
Funnel plot assessing publication bias among the included studies.

Publication bias was assessed using both funnel plots and Egger regression asymmetry test. The funnel plots demonstrated a symmetrical distribution of effect sizes, suggesting no significant visual indication of publication bias (Fig. 9). Additionally, Egger test yielded a *P*-value > .05, indicating no statistically significant evidence of publication bias across the included studies. These findings collectively suggest that the results of the meta-analysis are unlikely to be affected by small-study effects or selective reporting, thereby reinforcing the reliability and validity of the pooled estimates.

## 4. Discussion

Liver abscesses, characterized by localized collections of pus within the liver parenchyma, are a serious clinical condition with potentially life-threatening complications. Advances in diagnostic imaging and minimally invasive techniques have significantly transformed the management of this condition. Among these, PA and PCD have emerged as the primary therapeutic approaches due to their effectiveness and reduced invasiveness compared to surgical drainage.^[28,29]^ However, there remains ongoing debate regarding the optimal choice between these 2 methods, particularly in terms of success rates, time to clinical improvement, recurrence prevention, and safety profiles. PA, which involves a single-session needle aspiration, is often preferred for its simplicity and lower cost, making it suitable for smaller, uncomplicated abscesses. Conversely, PCD, which entails the placement of a catheter for continuous drainage, is considered advantageous in treating larger or multiloculated abscesses and cases prone to recurrence.^[30,31]^ This meta-analysis provides a comprehensive comparison of PA and PCD in the treatment of liver abscesses, focusing on treatment success rates, time to clinical improvement, recurrence rates, and complication profiles. The findings underscore the clinical advantages of PCD in several key areas while also emphasizing the safety and practicality of both approaches under specific circumstances.

The analysis demonstrated that PCD achieved significantly higher treatment success rates compared to PA, with a RR of 1.19 (95% CI [1.10, 1.29]). This reflects a 19% greater probability of resolving abscesses with PCD. The superiority of PCD is likely attributable to its ability to provide continuous and sustained drainage, which is particularly effective for large, multiloculated, or complex abscesses. In contrast, PA is limited by its single-session nature and often requires repeated procedures to achieve comparable outcomes. The heterogeneity observed among the included studies (*I*² = 73.3%, *P* < .001) highlights the impact of variability in abscess characteristics, procedural techniques, and patient factors on treatment outcomes. For example, larger and more complex abscesses may disproportionately benefit from the sustained drainage offered by PCD.^[32]^ Sensitivity analyses confirmed the robustness of these findings, reinforcing the conclusion that PCD is more effective in achieving higher success rates.

The pooled analysis of time to clinical improvement further supported the clinical superiority of PCD, with a weighted MD of −1.92 days (95% CI [−2.71, −1.13]). This finding suggests that PCD expedites recovery by nearly 2 days compared to PA, which has significant implications for reducing hospital stays and healthcare costs. The shorter recovery time with PCD can be attributed to its continuous drainage mechanism, which effectively reduces abscess volume and local inflammation more rapidly. Despite significant heterogeneity among studies (*I*^2^ = 91.1%, *P* < .001), sensitivity analyses confirmed the stability of the results, underscoring their reliability. The observed heterogeneity likely stems from differences in definitions of clinical improvement, patient demographics, and operator expertise. These findings highlight the potential of PCD to enhance patient outcomes and facilitate quicker return to normal activities.^[33]^

The analysis of recurrence rates 6 months after treatment revealed a significant advantage for PCD, with an RR of 0.44 (95% CI [0.26, 0.75]). This translates to a 56% reduction in the risk of recurrence compared to PA. The lower recurrence rates with PCD may be attributed to its more complete and sustained drainage of abscess contents, which minimizes residual infection and prevents reaccumulation. Unlike other outcomes, recurrence rates showed no significant heterogeneity among the included studies (*I*^2^ = 0.0%, *P* = .676), allowing the use of a fixed-effects model. The consistency of these results across studies supports the long-term efficacy of PCD in reducing the burden of recurrent liver abscesses.^[34]^

Both PA and PCD were found to have comparable complication rates, with an RR of 1.13 (95% CI [0.45, 2.85], *P* > .05). This finding highlights the similar safety profiles of the 2 procedures, providing clinicians with flexibility to base treatment decisions on other factors such as efficacy and patient preferences. The absence of significant heterogeneity (*I*^2^ = 0.0%, *P* = .868) further reinforces the reliability of this conclusion. The comparable safety profiles can be attributed to the minimally invasive nature of both techniques. Common complications, such as minor bleeding, catheter blockage, or infection, are generally manageable and do not significantly differ between the 2 modalities.

While PA is effective for smaller, uncomplicated abscesses due to its simplicity and cost-effectiveness, PCD excels in managing larger, more complex abscesses due to its sustained drainage capability. The shorter time to clinical improvement and lower recurrence rates with PCD underscore its potential as the preferred approach for more severe cases.^[35,36]^ The lack of significant differences in complication rates highlights the overall safety of both techniques, reinforcing their utility as first-line interventions for liver abscesses. Clinicians should consider factors such as abscess characteristics, patient comorbidities, and resource availability when selecting the most appropriate treatment strategy.

Our findings are consistent with those of a recent meta-analysis by Chuang et al, published in BMJ Open in 2023, which included 10 RCTs and conducted both conventional meta-analysis and trial sequential analysis.^[37]^ Similar to our study, their analysis showed that PCD significantly improved treatment success rates compared to percutaneous needle aspiration (RR = 1.16, 95% CI [1.07–1.25]) and led to faster clinical improvement (MD = –2.53 days). They further demonstrated reductions in time to abscess shrinkage and duration of intravenous antibiotic therapy in the PCD group, although no differences were observed in complication rates or mortality. These findings reinforce the overall superiority of PCD, particularly in managing large abscesses. Notably, the effect sizes reported in our study are slightly more pronounced (e.g., RR = 1.19 for success), potentially due to differences in study selection (we included 12 trials with broader geographic representation), definitions of outcomes, and the absence of trial sequential analysis in our methodology. Nonetheless, both studies contribute complementary evidence supporting the preferential use of PCD in appropriate clinical scenarios. In summary, this meta-analysis provides updated and comprehensive evidence favoring the use of percutaneous catheter drainage over PA for the treatment of liver abscess. PCD demonstrated superior outcomes in terms of treatment success, speed of clinical recovery, and recurrence prevention, with no significant increase in complications.

This study has several limitations that warrant consideration. First, significant heterogeneity was observed in some outcomes, particularly treatment success rates and time to clinical improvement, which may have been influenced by variations in study design, patient demographics, abscess characteristics, and procedural protocols. Second, the included studies often lacked standardized definitions for clinical improvement and recurrence, potentially introducing bias. Furthermore, publication bias, although not detected in the funnel plot analysis, cannot be completely excluded due to the inherent limitations of the included studies. Future research should focus on conducting well-designed, multicenter RCTs with standardized protocols to validate these findings. Emphasis should also be placed on exploring patient-specific factors, such as abscess size and underlying comorbidities, to refine treatment selection criteria. Long-term follow-up studies are needed to assess recurrence prevention and overall patient outcomes, further strengthening the evidence base for clinical guidelines.

## 5. Conclusions

This meta-analysis highlights the superior outcomes of PCD compared to PA for the treatment of liver abscesses, with higher success rates, shorter time to clinical improvement, and lower recurrence rates, while exhibiting comparable safety profiles. However, further high-quality, multicenter RCTs are needed to confirm these findings and strengthen the evidence base.

## Acknowledgments

We thank everyone who participated in this research.

## Author contributions

**Conceptualization:** Xu-Feng Li, Dan-Mei Pan.

**Data curation:** Dan-Mei Pan.

**Formal analysis:** Dan-Mei Pan.

**Investigation:** Dan-Mei Pan.

**Methodology:** Xu-Feng Li, Dan-Mei Pan.

**Resources:** Dan-Mei Pan.

**Software:** Dan-Mei Pan.

**Writing – original draft:** Dan-Mei Pan.

**Writing – review & editing:** Xu-Feng Li.

## References

[1] Kaplan GG Gregson DB Laupland KB. Population-based study of the epidemiology of and the risk factors for pyogenic liver abscess. Clin Gastroenterol Hepatol. 2004;2:1032–8. 15551257 10.1016/s1542-3565(04)00459-8

[2] Losie JA Lam JC Gregson DB Parkins MD. Epidemiology and risk factors for pyogenic liver abscess in the Calgary Health Zone revisited: a population-based study. BMC Infect Dis. 2021;21:939. 34507537 10.1186/s12879-021-06649-9 PMC8431851

[3] Lardière-Deguelte S Ragot E Amroun K. Hepatic abscess: diagnosis and management. J Visc Surg. 2015;152:231–43. 25770745 10.1016/j.jviscsurg.2015.01.013

[4] Ribeiro Da Costa R Andres A Huttner B. Pyogenic liver abscesses. Rev Med Suisse. 2020;16:1822–6. 32997454

[5] Chan KS H’Ng MWC Shelat VG. Is there a role for percutaneous needle aspiration in the multimodal management of pyogenic liver abscess? Ann Transl Med. 2023;11:306. 37404991 10.21037/atm-23-1385 PMC10316104

[6] Das S Shankar G Mohapatra V. Safety and efficacy of USG-guided catheter drainage in liver abscesses. Ann Afr Med. 2022;21:21–5. 35313400 10.4103/aam.aam_68_20 PMC9020638

[7] Priyadarshi RN Kumar R Anand U. Amebic liver abscess: clinico-radiological findings and interventional management. World J Radiol. 2022;14:272–85. 36160830 10.4329/wjr.v14.i8.272 PMC9453321

[8] Zhang S Xu Q Liu C Wu Z Chen Z Gu S. Management and prognostic prediction of pyogenic liver abscess in a Chinese tertiary hospital: percutaneous needle aspiration vs catheter drainage. PLoS One. 2024;19:e0315371. 39680538 10.1371/journal.pone.0315371 PMC11649130

[9] He S Yu J Wang H Chen X He Z Chen Y. Percutaneous fine-needle aspiration for pyogenic liver abscess (3–6 cm): a two-center retrospective study. BMC Infect Dis. 2020;20:516. 32677915 10.1186/s12879-020-05239-5 PMC7364546

[10] Goyal A Dhaliwal HS Nampoothiri RV. Percutaneous catheter drainage of uncomplicated amoebic liver abscess: prospective evaluation of a clinical protocol for catheter removal and the significance of residual collections. Abdom Radiol (NY). 2021;46:2855–64. 33469690 10.1007/s00261-021-02949-5

[11] Zerem E Hadzic A. Sonographically guided percutaneous catheter drainage versus needle aspiration in the management of pyogenic liver abscess. AJR Am J Roentgenol. 2007;189:W138–142. 17715080 10.2214/AJR.07.2173

[12] Kumar S Midha NK Ahari K. Role of pigtail catheter drainage versus percutaneous needle aspiration in the management of liver abscess: a retrospective analysis. Cureus. 2021;13:e20528. 35070562 10.7759/cureus.20528 PMC8767523

[13] Liu Y Li Z Liu A. Early percutaneous catheter drainage in protecting against prolonged fever among patients with pyogenic liver abscess: a retrospective cohort study. Ann Med. 2022;54:2269–77. 35975970 10.1080/07853890.2022.2110612 PMC9387318

[14] Shinmoto K Hiraoka E Horiuchi M. Impact of antibiotic timing relative to percutaneous aspiration on culture positivity rate and clinical outcomes: a retrospective study of patients with pyogenic liver abscess. J Infect Chemother. 2022;28:336–8. 34756828 10.1016/j.jiac.2021.10.007

[15] Page MJ McKenzie JE Bossuyt PM. The PRISMA 2020 statement: an updated guideline for reporting systematic reviews. BMJ. 2021;372:n71. 33782057 10.1136/bmj.n71 PMC8005924

[16] Higgins JP Altman DG Gøtzsche PC. The Cochrane Collaboration’s tool for assessing risk of bias in randomised trials. BMJ. 2011;343:d5928. 22008217 10.1136/bmj.d5928 PMC3196245

[17] Abusedera MA El-Badry AM. Percutaneous treatment of large pyogenic liver abscess. Egypt J Radiol Nucl Med. 2014;45:109–15.

[18] Ahmed M Alam J Hussain S Aslam M. Prospective randomized comparative study of percutaneous catheter drainage and percutaneous needle aspiration in the treatment of liver abscess. ANZ J Surg. 2021;91:E86–90. 33244881 10.1111/ans.16461

[19] Gajera D Shah M Makwana N Rathwa A. Comparative study of percutaneous catheter drainage versus percutaneous needle aspiration for liver abscess. Int J Health Sci. 2022;6(S6):282–8.

[20] Gupta SS Singh O Sabharwal G Hastir A. Catheter drainage versus needle aspiration in management of large (>10 cm diameter) amoebic liver abscesses. ANZ J Surg. 2011;81:547–51. 22295386 10.1111/j.1445-2197.2010.05494.x

[21] Kulhari M Mandia R. Prospective randomized comparative study of pigtail catheter drainage versus percutaneous needle aspiration in treatment of liver abscess. ANZ J Surg. 2019;89:E81–6. 30362216 10.1111/ans.14917

[22] Rajak CL Gupta S Jain S Chawla Y Gulati M Suri S. Percutaneous treatment of liver abscesses: needle aspiration versus catheter drainage. AJR Am J Roentgenol. 1998;170:1035–9. 9530055 10.2214/ajr.170.4.9530055

[23] Singh O Gupta S Moses S Jain DK. Comparative study of catheter drainage and needle aspiration in management of large liver abscesses. Indian J Gastroenterol. 2009;28:88–92. 19907956 10.1007/s12664-009-0032-1

[24] Singh P Tapasvi C Kaur R Aggarwal S Nagpal N Kaur R. Prospective randomized comparison of ultrasound-guided percutaneous needle aspiration with percutaneous catheter drainage of liver abscesses. J Med Sci. 2019;39:67–73.

[25] Singh S Chaudhary P Saxena N Khandelwal S Poddar DD Biswal UC. Treatment of liver abscess: prospective randomized comparison of catheter drainage and needle aspiration. Ann Gastroenterol. 2013;26:332–9. 24714320 PMC3959473

[26] Surya M Bhoil R Sharma YP. Study of ultrasound-guided needle aspiration and catheter drainage in the management of liver abscesses. J Ultrasound. 2020;23:553–62. 32221809 10.1007/s40477-020-00440-3 PMC7588573

[27] Yu SC Ho SS Lau WY. Treatment of pyogenic liver abscess: prospective randomized comparison of catheter drainage and needle aspiration. Hepatology. 2004;39:932–8. 15057896 10.1002/hep.20133

[28] Vakamacawai E McCaig E Waqainabete I Cox MR. Amoebic liver abscesses in Fiji: epidemiology, clinical presentation and comparison of percutaneous aspiration and percutaneous catheter drainage. World J Surg. 2020;44:665–72. 31712845 10.1007/s00268-019-05274-7

[29] Xu S Shi BQ Chao LM Tan YS Zhang XJ. Prognostic nomogram for the combination therapy of percutaneous catheter drainage and antibiotics in pyogenic liver abscess patients. Abdom Radiol (NY). 2020;45:393–402. 31797027 10.1007/s00261-019-02359-8

[30] Sahu V Pipal DK Singh Y. Epidemiology, clinical features, and outcome of liver abscess: a single-center experience. Cureus. 2022;14:e29812. 36337811 10.7759/cureus.29812 PMC9621470

[31] Priyadarshi RN Prakash V Anand U Kumar P Jha AK Kumar R. Ultrasound-guided percutaneous catheter drainage of various types of ruptured amebic liver abscess: a report of 117 cases from a highly endemic zone of India. Abdom Radiol (NY). 2019;44:877–85. 30361869 10.1007/s00261-018-1810-y

[32] Ballard DH Flanagan ST Brown RW Vea R Ahuja C D’Agostino HB. Paired drainage catheter insertion: feasibility of placing two catheters within the same complex abscess cavity as a primary and salvage percutaneous drainage technique. Acad Radiol. 2020;27:e1–9. 31031185 10.1016/j.acra.2019.03.010 PMC6814525

[33] Liu CH Gervais DA Hahn PF Arellano RS Uppot RN Mueller PR. Percutaneous hepatic abscess drainage: do multiple abscesses or multiloculated abscesses preclude drainage or affect outcome? J Vasc Interv Radiol. 2009;20:1059–65. 19560374 10.1016/j.jvir.2009.04.062

[34] Ghosh JK Goyal SK Behera MK. Efficacy of aspiration in amebic liver abscess. Trop Gastroenterol. 2015;36:251–5. 27509703 10.7869/tg.299

[35] Ming Y Wei H Zhang Y. The effect of contrast-enhanced ultrasound via vessels and surgical drains guidance percutaneous catheter drainage in the treatment of pyogenic liver abscess. Curr Med Imaging. 2024;20:e15734056261616. 38454768 10.2174/0115734056261616231224161652

[36] Haider SJ Tarulli M McNulty NJ Hoffer EK. Liver abscesses: factors that influence outcome of percutaneous drainage. AJR Am J Roentgenol. 2017;209:205–13. 28504550 10.2214/AJR.16.17713

[37] Lin JW Chen CT Hsieh MS. Percutaneous catheter drainage versus percutaneous needle aspiration for liver abscess: a systematic review, meta-analysis and trial sequential analysis. BMJ Open. 2023;13:e072736. 10.1136/bmjopen-2023-072736 PMC10387661 37518084

